# Nurses’ Perception of Artificial Intelligence-Driven Monitoring Systems for Enhancing Compliance With Infection Prevention and Control Measures in Al-Ahsa, Saudi Arabia

**DOI:** 10.7759/cureus.82943

**Published:** 2025-04-24

**Authors:** Sahbanathul Missiriya Jalal, Suhail Hassan Jalal, Kamilah Essa Alasmakh, Zahraa Hussain Alnasser, Wadiah Yousef Alhamdan, Abeer Abbas Alabdullatif

**Affiliations:** 1 Nursing Department, College of Applied Medical Sciences, King Faisal University, Hofuf, SAU; 2 Pharmacy Department, Jaya College of Pharmacy, The Tamil Nadu Dr. M.G.R. Medical University, Chennai, IND; 3 Critical Care Unit, Prince Sultan Cardiac Center, Hofuf, SAU; 4 Intensive Care Unit, Al Omran General Hospital, Al Omran, SAU; 5 Nursing Department, Al-Moosa College of Health Science, Al Mubarraz, SAU

**Keywords:** artificial intelligence, compliance, healthcare-associated infections, infection prevention and control, monitoring systems, nurses, perception

## Abstract

Background

Healthcare-associated infections (HCAIs) represent a major risk to patient safety, increasing morbidity, mortality, and costs. Effective infection prevention and control (IPC) compliance is crucial, but nurse adherence remains inconsistent, necessitating innovative solutions such as artificial intelligence (AI)-driven monitoring. However, the success of such technologies heavily relies on the perceptions and acceptance of frontline healthcare workers, particularly nurses. This study aimed to determine the nurses’ perception of AI-driven monitoring in improving IPC compliance in selected hospitals.

Methodology

A cross-sectional study was conducted among nurses working at a public hospital in Al-Ahsa, Saudi Arabia. Computer-generated numbers randomly selected 246 nurses. A structured, self-administered questionnaire was used to gather data on demographics, knowledge, perceptions, and perceived barriers to AI-driven monitoring in IPC practices. Descriptive statistics were utilized for continuous variables, while inferential statistics, such as chi-square, were used for categorical variables to analyse the results.

Results

Out of 246 nurses, 183 (74.4%) had average knowledge about AI applications in IPC practices. The overall mean knowledge score regarding AI-based IPC measures was 17.00 ± 3.97 out of 20, which showed that most nurses had moderate knowledge, but some domains scored well. Regarding perception about AI-driven monitoring IPC practices, many nurses had a positive attitude. However, insufficient training, financial limitations, and limited organizational support are perceived as the most critical barriers. There was a significant association found between the level of knowledge and age, highest educational qualification, job role, and AI technology-based IPC training (p < 0.05). Nurses expressed willingness to adopt AI systems if adequate training and support were ensured.

Conclusion

AI-driven monitoring may enhance IPC compliance among nurses if barriers are addressed, helping to reduce HCAIs and improve patient safety. Its success depends on addressing key barriers such as training, infrastructure, and stakeholder support. These findings can guide policymakers and healthcare leaders in effectively adopting AI-based IPC solutions.

## Introduction

Healthcare-associated infections (HCAIs) threaten patient safety, leading to increased morbidity and mortality and raising healthcare costs worldwide. Nurses, as primary caregivers, play a pivotal role in implementing infection prevention and control (IPC) measures to reduce these risks. However, ensuring consistent compliance with IPC protocols remains a challenge in many healthcare settings [1].​

According to the World Health Organization (WHO), over 24% of patients affected by healthcare-associated sepsis and 52.3% of those treated in intensive care units (ICUs) die annually [2]. In the United States, the Centers for Disease Control and Prevention (CDC) reported that 3.2% of hospitalized patients experience HCAIs. Similarly, in the European Union/European Economic Area (EU/EEA), the prevalence rises to 6.5%. Globally, the actual prevalence is likely higher, though comprehensive data remain limited [3].

A point prevalence survey conducted across 99 acute care hospitals in 2022 found that 10.1% of patients had at least one HCAI. The most common types were bloodstream infections (18.9%), urinary tract infections (17.1%), SARS-CoV-2 infections (17.0%), pneumonia and lower respiratory tract infections (16.7%), and surgical site infections (11.0%) [4]. These reports highlight how important it is to implement strong IPC initiatives globally to lessen the negative effects of HCAIs on patient health and healthcare systems. In Saudi Arabia, studies have explored the infection prevention climate among nurses. For instance, research conducted in two general hospitals in Riyadh province assessed staff nurses’ perceptions of the infection prevention climate and its predictors, highlighting the importance of organizational context in influencing IPC programs [5].

HCAIs threaten patient safety, leading to increased morbidity and mortality and raising healthcare costs worldwide. Nurses, as primary caregivers, play a pivotal role in implementing IPC measures to reduce these risks. However, ensuring consistent compliance with IPC protocols remains a challenge in many healthcare settings [1].​

According to the WHO, over 24% of patients affected by healthcare-associated sepsis and 52.3% of those treated in ICUs die annually [2]. In the United States, the CDC reported that 3.2% of hospitalized patients experience HCAIs. Similarly, in the EU/EEA, the prevalence rises to 6.5%. Globally, the actual prevalence is likely higher, though comprehensive data remain limited [3].

A point prevalence survey conducted across 99 acute care hospitals in 2022 found that 10.1% of patients had at least one HCAI. The most common types were bloodstream infections (18.9%), urinary tract infections (17.1%), SARS-CoV-2 infections (17.0%), pneumonia and lower respiratory tract infections (16.7%), and surgical site infections (11.0%) [4]. These reports highlight how important it is to implement strong IPC initiatives globally to lessen the negative effects of HCAIs on patient health and healthcare systems. In Saudi Arabia, studies have explored the infection prevention climate among nurses. For instance, research conducted in two general hospitals in Riyadh province assessed staff nurses’ perceptions of the infection prevention climate and its predictors, highlighting the importance of organizational context in influencing IPC programs [5].

Artificial intelligence (AI) has become a transformative shift in healthcare, providing groundbreaking solutions to enhance IPC compliance, as it is essential for monitoring and forecasting outbreaks of infectious diseases. AI-powered systems can track adherence to key IPC measures such as hand hygiene protocols and PPE use while providing real-time feedback and actionable insights. A systematic review examining AI-powered tools for monitoring, identifying, and managing HAIs emphasized their increasing use and encouraging prospects in this area [6]. Significantly, AI integration has demonstrated significant success in enhancing IPC practices, particularly in critical care environments.

Studies have demonstrated AI's effectiveness in accurately predicting and mitigating HCAIs, enhancing real-time patient monitoring, and automating sterilization processes. These advancements improve patient outcomes and resource optimization in ICUs [7]. Furthermore, AI technologies have been applied to train and report infection prevention measures in critical wards. AI-integrated handwashing devices can provide daily training and objectively assess the efficacy of IPC measures, addressing common barriers to proper hand hygiene such as time constraints and lack of knowledge [8].

In addition to monitoring and training, AI has been utilized for automatic video auditing with real-time feedback, significantly improving the quality and quantity of hand hygiene events in hospital settings. This approach not only enhances compliance but also fosters a culture of accountability among healthcare workers [9]. Despite the technological advancements, the successful integration of AI in clinical practice largely depends on the readiness, acceptance, and perceptions of healthcare professionals. Nurses' perceptions toward AI-driven systems can significantly influence the adoption, utilization, and sustainability of such technologies in infection control efforts. Understanding their viewpoints is essential to tailor implementation strategies, address concerns, and optimize the benefits of AI integration.

In Saudi Arabia, where digital transformation in healthcare is gaining momentum under the Vision 2030 framework, assessing the receptiveness of nurses toward AI tools is particularly relevant. In regions such as Al-Ahsa, where both public and private healthcare sectors are expanding, evaluating the local context is crucial to inform policy and practice [10].

This study aimed to explore the perceptions, awareness, and attitudes of nurses toward the use of AI-driven monitoring systems for enhancing compliance with IPC measures in hospitals. The findings will contribute valuable insights into the facilitators and barriers influencing technology acceptance and guide effective implementation of AI-based solutions in infection prevention.

## Materials and methods

Study design

Cross-sectional research was conducted to assess the AI-driven monitoring with the compliance of IPC practices among nurses. Ethical approval was obtained from the Ethics Committee at King Fahad Hospital, Al-Ahsa, Saudi Arabia (approval number: 29A-41-2024). Before their participation, all participants signed an informed consent form in either Arabic or English. Throughout the study, ethical considerations and confidentiality of participants’ information were strictly maintained using an anonymized data collection tool.

Study setting and participants

This study was conducted at three major healthcare facilities in Al-Ahsa, Saudi Arabia: King Fahad Hospital, Bin Jalawi Hospital, and Al Omran Hospital. These hospitals are among the primary healthcare providers in the region, catering to a diverse patient population and employing a substantial number of nursing staff. The study focused on registered nurses working in various departments, including intensive care units (ICUs), surgical wards, medical wards, emergency departments, and outpatient clinics. Registered nurses, including both genders with at least one year of clinical experience, were included in the study, while those nurses on extended leave or unwilling to participate were excluded from the study.

Study sampling

The sample size for this study was calculated using the single population proportion formula, based on the assumption that 50% of the target population had adequate knowledge and good infection prevention practices, as indicated by findings from a previous study conducted by Ayat et al. [11]. This conservative estimate maximizes sample size and ensures statistical reliability. A 5% margin of error and a 95% confidence level were applied to enhance precision and confidence in the results. Considering the finite population of 385 nurses, the formula was adjusted accordingly to reflect the actual population size. After accounting for exclusions due to incomplete data or non-response, a final sample of 246 nurses was selected. To minimize selection bias, simple random sampling was used, and participants were identified using a computer-generated random number system, ensuring each eligible nurse had an equal chance of being included in the study.

Data collection

A structured questionnaire was used to collect the data. This questionnaire is an original tool, and it has been evaluated by a panel of medical and nursing experts to validate the tool. A pilot study has also been conducted to improve the tool. The questionnaire demonstrated high reliability (Cronbach’s α = 0.916). The structured questionnaires consisted of the following parts: 1) demographic variables of nurses; 2) awareness and knowledge; 3) perceptions and attitudes; and 4) perceived barriers of AI-driven monitoring systems for enhancing compliance with IPC measures. Approximately 15-20 minutes are needed to complete the tool. Before distribution, participants received an introduction outlining the study’s objectives, along with assurances of anonymity, confidentiality, and voluntary participation (no financial incentives were provided). They were informed that their participation in the study was voluntary without any financial support. Written informed consent was obtained from all the participants before the data collection.

Part 1: Demographic Variables

The demographic variables of the nurses included age, gender, highest educational qualification, job role, years of experience, nationality, and AI technology-based IPC training.

Part 2: Knowledge About AI Applications in IPC Measures

The second part of the tool focused on assessing nurses’ knowledge and understanding of AI applications in enhancing compliance with IPC measures. It consisted of 20 multiple-choice questions, each designed to evaluate core concepts related to AI-driven monitoring systems and their role in IPC. For scoring, each correct answer was awarded one point, while incorrect answers received zero points, resulting in a possible score range of 0-20. Based on the total score, knowledge levels were categorized as follows: Score less than 10 as poor knowledge, score between 10 and 14 as average knowledge, and score of 15 and above as good knowledge. This classification helped in identifying knowledge gaps and tailoring future training initiatives accordingly.

Part 3: Perceptions About AI Applications in IPC Measures

This section included five statements designed to evaluate nurses' beliefs about the improvement in compliance, role complementarity, clarity and usefulness, ethical considerations, privacy of using AI monitoring in clinical settings, and comfort and willingness to use AI applications in IPC measures. Responses were captured on a 5-point Likert scale ranging from strongly agree to strongly disagree. The score five was provided to strongly agree; score four was given to agree; score three for neutral; score two for disagree; and score one was provided to strongly disagree. The total possible score range is from five to 25 (minimum: 5 statements × 1 = 5; maximum: 5 × 5 = 25). According to the score, the categorization are follows; the positive perception (19-25) is consistent agreement (mostly 4-5 ratings); neutral perception (13-18) is mixed or indifferent responses (mostly 3 ratings); and negative perception (5-12) is consistent disagreement (mostly 1-2 ratings).

Part 4: Perceived Barriers

This part addressed potential challenges to the implementation of AI systems, with a closed-ended response checklist of “Yes,” “No," and “I do not know.” Participants were asked to identify key obstacles such as lack of training and awareness, technological limitations, privacy and ethical concerns, resistance to change, cost and resource constraints, and limited organizational support.

Data analysis

Statistical analysis was performed with Statistical Package for Social Sciences (SPSS) for Windows (version 21.0; International Business Machines (IBM) Corporation, Armonk, NY). The statistical significance level was set at p < 0.05. Descriptive statistics, such as frequency and percentages, were used for categorical variables, and the mean and standard deviation (SD) were used for continuous variables. Descriptive statistics were employed to summarize continuous variables, such as age and years of experience, using means and standard deviations. Inferential statistics, such as chi-square tests, were applied to examine associations between the level of knowledge and demographic factors.

## Results

Demographic variables

The demographic characteristics of the study participants were analysed and are shown in Table 1. Among the total of 246 nurses who participated in the study, most participants (103, 41.8%) were aged 21-30 years, followed by those aged 31-40 years (89, 36.2%) and above 40 years (54, 22%). In terms of gender distribution, most participants (188, 76.4%) were female, while 58 (23.6%) participants were male. Regarding educational qualifications, the highest proportion of participants (152, 61.8%) held a graduate degree, followed by 59 (24%) diploma holders and 35 (14.2%) with a postgraduate qualification. Regarding job role, more than half of the participants (135, 54.9%) were staff nurses. In terms of professional experience, 98 (39.8%) nurses had between one and five years of experience, 86 (35%) had 6-10 years, and 62 (25.2%) had more than 10 years of work experience. Regarding nationality, the majority (167, 67.9%) were non-Saudi nationals, whereas 79 (32.1%) were Saudi nationals. When asked about AI-based IPC training, only 81 (32.9%) had received such training.

**Table 1:** Frequency distribution of demographic variables of the nurses (n = 246)

Variables	Category	Number	Percentage
Age (years)	21-30 Years	103	41.8
	31-40 Years	89	36.2
	More than 40 Years	54	22
Gender	Male	58	23.6
	Female	188	76.4
Educational Qualification (Highest)	Diploma	59	24
	Graduate	152	61.8
	Postgraduate Level	35	14.2
Job Role	Staff Nurse	135	54.9
	Senior Charge Nurse	78	31.7
	Head Nurse	33	13.4
Years of Experience	1-5 Years	98	39.8
	6-10 Years	86	35
	> 10 Years	62	25.2
Nationality	Saudi	79	32.1
	Non-Saudi	167	67.9
AI-Based IPC Training	Yes	81	32.9
	No	165	67.1

Knowledge level of nurses regarding AI applications in IPC measures

**Figure 1:**
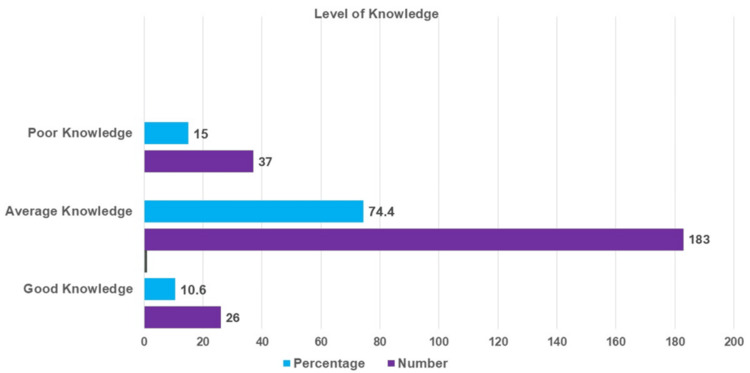
Frequency distribution of the knowledge level of nurses about AI applications in IPC measures IPC: infection prevention and control

The frequency distribution of the knowledge level of nurses about AI applications in IPC measures was shown in Figure 1. Among the 246 nurses surveyed, many of them (183, 74.4%) demonstrated average knowledge, and 37 (15%) showed poor knowledge. A smaller portion, 26 (10.6%) nurses, exhibited good knowledge. The descriptive statistics of nurses’ knowledge scores across various domains of AI-driven monitoring IPC practices are shown in Table 2. The questionnaire consisted of 20 items distributed across five key subscales: The first domain included five items, with participants scoring a mean of 4.53 ± 0.99, indicating a high level of awareness in general IPC concepts. About the hand hygiene monitoring using AI comprised four items, with a mean score of 2.26 ± 0.90, suggesting moderate knowledge in this critical area. Regarding PPE usage monitoring by AI was assessed through five items, resulting in a mean of 2.96 ± 1.40, reflecting variability in understanding and practices related to PPE. The surface disinfection (three items) yielded a mean of 1.59 ± 1.02, indicating relatively lower knowledge in this area. The equipment sterilization (three items) had a mean score of 2.23 ± 1.25, showing moderate knowledge. The overall mean knowledge score across all 20 items was 17.00 ± 3.97, indicating a moderate to high level of knowledge regarding IPC measures using AI. While this suggests that participants generally had a solid understanding, certain subdomains, particularly surface disinfection and hand hygiene, showed areas where further improvement is needed.

**Table 2:** Mean scores of nurses’ knowledges about AI applications in IPC measures per subscale (n = 246) IPC: infection prevention and control; PPE: personal protective equipment; SD: standard deviation

Knowledge Questionnaire	Number of items	Range	Mean (± SD)
IPC common principles	5	0–5	4.53 (0.99)
Hand hygiene	4	0–4	2.26 (0.9)
PPE usage	5	0–5	2.96 (1.4)
Surface disinfection	3	0–3	1.59 (1.02)
Equipment sterilization	3	0–3	2.23 (1.25)
Total	20	0–20	17 (3.97)

Nurses’ perception of AI-driven compliance monitoring systems

**Figure 2:**
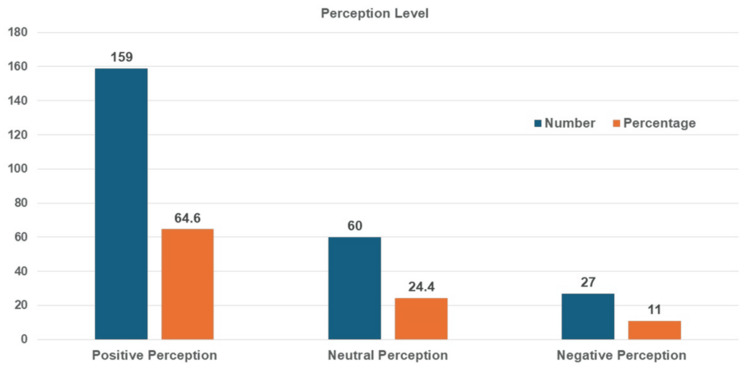
Frequency distribution of nurses’ perceptions about AI applications in IPC measures IPC: infection prevention and control

The perception level of nurses toward AI-driven monitoring for IPC was illustrated in Figure 2. Most of the nurses (159, 64.6%) showed positive perception, 60 (24.4%) nurses showed neutral perception, and 27 (11%) nurses expressed a negative perception about AI-driven monitoring for enhancing the compliance of IPC practices.

Table 3 summarizes the responses of nurses regarding their perceptions and attitudes toward AI-driven monitoring systems in enhancing IPC practices. The items were rated on a 5-point Likert scale, ranging from strongly agree to strongly disagree. Regarding the AI system improved compliance with IPC, 69 (28%) nurses strongly agreed, and 88 (35.8%) agreed. About AI systems are designed to support nurses in IPC, 87 (35.4%) nurses strongly agreed, and 71 (28.9%) agreed. Meanwhile, 94 (38.2%) nurses agreed, and 62 (25.2%) were neutral towards clear and actionable feedback given by AI monitoring on IPC. Regarding AI monitoring respects privacy and confidentiality, 96 (39%) nurses strongly agreed, and 15 (6.1%) nurses strongly disagreed. Around 81 (32.9%) nurses strongly agreed that comfort while using AI technologies for IPC.

**Table 3:** Frequency distribution of nurses’ perceptions about AI applications in IPC measures (n = 246) N: number; %: percentage; IPC: infection prevention and control

Nurse Perception on AI-Driven Monitoring	Strongly Agree (5)	Agree (4)	Neutral (3)	Disagree (2)	Strongly Disagree (1)
	N (%)	N (%)	N (%)	N (%)	N (%)
The AI system improved my compliance with IPC.	69 (28)	88 (35.8)	54 (22)	28 (11.4)	7 (2.8)
AI systems are designed to support, nurses in IPC.	87 (35.4)	71 (28.9)	56 (22.8)	22 (8.9)	10 (4)
The system provided clear and actionable feedback.	59 (24)	94 (38.2)	62 (25.2)	22 (8.9)	9 (3.7)
AI monitoring systems respect the privacy and confidentiality of both patients and healthcare staff.	96 (39)	63 (25.6)	48 (19.5)	24 (9.8)	15 (6.1)
I feel comfortable using AI technologies to assist in IPC.	81 (32.9)	69 (28)	60 (24.4)	28 (11.4)	8 (3.3)

Perceived barriers

Table 4 presents the distribution of nurse responses to a series of statements designed to identify perceived challenges in adopting AI technologies in IPC. Half of the nurses (129, 52.4%) agreed that they have not received adequate training on AI systems. Approximately 109 (44.3%) nurses identified technical problems as a significant barrier, 84 (34.1%) did not see it as a concern, and 53 (21.6%) were uncertain. Among them, 103 (41.9%) participants believed that there is a chance of ethical challenges with AI in IPC, such as surveillance and confidentiality. Only 76 (30.9%) felt that nurses are reluctant to adopt AI technologies. Financial limitations were perceived as a key barrier by 157 (63.8%) respondents. Nearly half (122, 49.6%) felt that there was limited institutional support to introduce AI systems in their workplace.

**Table 4:** Frequency distribution of perceived barriers about AI applications in IPC measures (n = 246) N: number; %: percentage; IPC: infection prevention and control

Perceived Barrier Statement	Yes	No	I Do not Know
	N (%)	N (%)	N (%)
Nurses have not received adequate training on AI systems used in infection control.	129 (52.4)	88 (35.8)	29 (11.8)
Technical issues are a major concern with AI implementation.	109 (44.3)	84 (34.1)	53 (21.6)
Chance for ethical implications of using AI in IPC.	103 (41.9)	79 (32.1)	64 (26)
Nurses are reluctant to adopt new AI technologies in IPC.	76 (30.9)	153 (62.2)	17 (6.9)
Implementing AI systems would require financial resources that may not be currently available.	157 (63.8)	65 (26.4)	24 (9.8)
There is limited organizational support to introduce AI in my workplace.	122 (49.6)	76 (30.9)	48 (19.5)

Association between knowledge levels and demographic variables

Table 5 presents the relationship between selected demographic and professional variables and nurses’ knowledge of AI-based IPC practices. A significant association was found between age and knowledge level (χ² = 33.73, df = 4, p < 0.001). Nurses aged 31-40 years showed a higher proportion of good knowledge, with an estimated 95% CI: 58%-70%, compared to other age groups. There was no significant relationship between gender and knowledge level (χ² = 2.47, df = 2, p = 0.29), with both male and female nurses showing comparable proportions of adequate knowledge (male: 46%, 95% CI: 38%-54%; female: 49%, 95% CI: 43%-55%). A statistically significant association was found between the highest educational qualification and knowledge level (χ² = 20.42, df = 4, p = 0.00041). Postgraduate nurses demonstrated a notably higher proportion of good knowledge (64%, 95% CI: 56%-72%) compared to those with a diploma (37%, 95% CI: 28%-46%) or graduate degrees (45%, 95% CI: 39%-51%). Job role was also significantly associated with knowledge level (χ² = 15.03, df = 4, p = 0.00464). Nurse educators and supervisors had higher levels of knowledge (educators: 68%, 95% CI: 54%-80%) than staff nurses (42%, 95% CI: 36%-48%). Nationality did not show a significant association with knowledge levels (χ² = 1.55, df = 2, p = 0.461), with both Saudi and non-Saudi nurses showing similar distributions of knowledge. Importantly, receiving AI-based IPC training was strongly associated with higher knowledge levels (χ² = 17.63, df = 2, p = 0.0001). Trained nurses demonstrated substantially better knowledge (71%, 95% CI: 62%-78%) than those who had not received training (41%, 95% CI: 35%-47%).

**Table 5:** Association between knowledge level and selected demographic variables (n = 246) * Significant; NS: nonsignificant; IPC: infection prevention and control

Variables	Category	Good Knowledge	Moderate Knowledge	Poor Knowledge	Chi-Square Tests		
		(n = 37)	(n = 183)	(n = 26)	X^2^	df	p value
Age (Years)	21-30 Years	10	79	14	33.73	4	0.00001*
	31-40 Years	6	72	11			
	> 40 Years	21	32	1			
Gender	Male	6	45	7	2.47	2	0.29 NS
	Female	37	138	19			
Educational Qualification (Highest)	Diploma	5	48	6	20.42	4	0.00041*
	Graduate	18	117	17			
	Postgraduate	14	18	3			
Nursing Role	Staff Nurse	14	103	18	15.03	4	0.00464*
	Charge Nurse	21	53	4			
	Head Nurse	2	27	4			
Years of Experience	1-5 Years	10	74	14	25.28	4	4.409 NS
	6-10 Years	6	71	9			
	> 10 Years	21	38	3			
Nationality	Saudi	9	60	10	1.55	2	0.461 NS
	Non-Saudi	28	123	16			
AI-Based IPC Training	Yes	22	56	3	17.63	2	0.0001*
	No	15	127	23			

## Discussion

The findings of this study provide valuable insights into nurses' knowledge, perceptions, and perceived barriers regarding AI-driven IPC practices. Most of the nurses (183, 74.4%) demonstrated average knowledge, with only 26 (10.6%) exhibiting good knowledge, suggesting a need for enhanced education and training on AI applications in IPC. Similar findings were reported by Wang et al., who noted that nurses often have limited awareness of AI’s role, despite its growing adoption in clinical settings [12].

The present study revealed significant associations between knowledge levels and age, education, job role, and prior AI-based IPC training. Younger nurses (21-30 years) had lower knowledge levels compared to older, more experienced nurses, which aligns with findings by the study done by Kotp et al. on age and experience as predictors of AI readiness among nurses, where clinical experience enhances familiarity with technological advancements [13]. In addition, nurses with postgraduate qualifications exhibited better knowledge, reinforcing the importance of higher education in adopting AI-driven healthcare innovations, as highlighted by research conducted on essential competencies of nurses working with AI-driven lifestyle monitoring in long-term care [14]. The lack of significant association between gender and knowledge contradicts some prior studies, carried out on gender differences in technology adoption among Saudi nurses, in which gender disparities were present in technology adoption among healthcare professionals [15]. However, the strong link between prior AI training and knowledge levels underscores the necessity of structured training programs, as emphasized in another study [16].

In the current study, a majority of 159 (64.6%) nurses had positive perceptions of AI-driven IPC monitoring, viewing it as a supportive tool that enhances compliance. This aligns with findings of a study on exploring nurses' awareness and attitudes toward artificial intelligence: implications for nursing practice, which reported that nurses appreciate AI for reducing human error in nursing practices [17]. However, concerns about privacy and ethical challenges were noted. A similar review on ethical considerations in the use of AI and machine learning in healthcare was done; it highlighted that AI technologies in a new era of personalized data-driven healthcare need to be emphasized for patient well-being and equity [18]. In our study, overall, the responses indicated a generally positive attitude among nurses toward AI-driven monitoring systems, particularly in terms of compliance support, comfort, and ethical trust. However, a noteworthy portion remains neutral, particularly regarding feedback clarity and privacy, suggesting areas for further education and system refinement.

The current study identified several barriers, including inadequate training, as reported by 129 (52.4%) nurses. Around 109 (44.3%) nurses reported technical issues as barriers, 157 (63.8%) reported financial constraints, and 122 (49.6%) reported limited institutional support as barriers. These findings resonate with the study carried out on barriers to AI implementation in Saudi hospitals by Saeed et al. in the year 2023, who identified resource limitations as a major obstacle to AI integration in healthcare [19]. Additionally, 103 (41.9%) nurses reported ethical concerns, and 76 (30.9%) nurses reported reluctance to adopt AI as perceived barriers, which supported arguments of Ahmed et al. in the year 2023, that resistance to change remains a challenge in digital health transformation [20].

The strength of this study included a diverse sample of nurses with varied age groups, educational backgrounds, job roles, and experience levels, enhancing the generalizability of findings. This aligns with recommendations by Doody et al. on ensuring representative samples in nursing research [21]. Real‐time patient monitoring and alert systems powered by AI are effective in enhancing infection detection and patient outcomes, which was supported by a literature review on enhancing infection control in ICUs through AI [22]. The study highlighted practical challenges (training deficits, financial constraints, ethical concerns), offering actionable insights for policymakers, consistent with the priorities identified by WHO for AI implementation in healthcare [23]. This study focuses on actionable barriers (e.g., institutional support, training needs), which were directly supported by the research [24]. However, this study design limits the ability to establish causal relationships. Longitudinal studies would be needed to confirm the long-term effects of AI systems and training on compliance.

To improve AI adoption in IPC, healthcare institutions should prioritize targeted training programs, as suggested in a study on AI in nursing for technological benefits to nurses' mental health and patient care quality [25]. Multi-center studies should compare barriers across institutions to identify universal vs. context-specific challenges. Addressing the financial and infrastructural barriers is crucial, as noted by research done by Ramezani et al. [26]. Furthermore, fostering a positive perception through transparent communication about AI benefits and ethical safeguards, as recommended in a literature review, can enhance acceptance [27,28].

## Conclusions

While nurses generally have moderate knowledge and positive perceptions of AI in IPC, significant gaps and barriers remain. Addressing these through education, institutional support, and ethical considerations will be essential for successful AI integration in infection control practices. The integration of AI into IPC practices holds immense potential for improving patient safety and reducing HCAIs. However, its success depends on addressing the identified knowledge gaps, fostering positive perceptions, and overcoming practical barriers. By adopting a strategic approach that combines education, policy reform, and technological investment, healthcare institutions can harness the full benefits of AI while mitigating its challenges. This study contributes to the growing body of literature on AI in nursing and provides a foundation for future research and policymaking in this evolving field.
